# 4,4′-diaponeurosporene, a C_30_ carotenoid, effectively activates dendritic cells *via* CD36 and NF-κB signaling in a ROS independent manner

**DOI:** 10.18632/oncotarget.9800

**Published:** 2016-06-02

**Authors:** Haofei Liu, Wenwen Xu, Xiaojing Chang, Tao Qin, Yinyan Yin, Qian Yang

**Affiliations:** ^1^ Key Laboratory of Animal Physiology and Biochemistry, Ministry of Agriculture, Nanjing Agricultural University, Nanjing, Jiangsu, People's Republic of China

**Keywords:** 4, 4′-diaponeurosporene, C_30_ carotenoids, dendritic cells, CD36, Immunology and Microbiology Section, Immune response, Immunity

## Abstract

Carotenoids could be divided into C_30_ carotenoids and C_40_ carotenoids. The immune functions of C_40_ carotenoids had been extensively researched, while those of C_30_ carotenoids still remain unclear. In this study, the immune functions of a biosynthetic C_30_ carotenoid, 4,4′-diaponeurosporene (Dia), were identified on dendritic cells (DCs). DCs treated with 1 μM Dia for 24 h showed morphologic and phenotypic characteristics of mature state and had an increased production of IL-6, IL-10, IL-12p70 and TNFα, while β-carotene had a suppressive effect on DCs maturation. Moreover, Dia promoted antigen uptake of DCs *in vitro* and increased the quantity of antigen loaded DCs in mesenteric lymph nodes (MLN). Dia-treated DCs also had an enhanced ability to stimulate T cell proliferation and Th1 polarization. Further researches showed that Dia activated DCs via CD36 as well as ERK, JNK, and NF-κB signals in a reactive oxygen species (ROS) independent manner.

## INTRODUCTION

The carotenoids, a subfamily of the isoprenoids, are among the most widespread of natural substances [1], and play important biological roles as accessory light-harvesting components of photosynthetic systems, photoprotecting antioxidants, and regulators of membrane fluidity [2]. Carotenoids could be divide into two categories, C_30_ carotenoids and C_40_ carotenoids, according to the molecular structure. The immune functions of many C_40_ carotenoids, such as β-carotene, lycopene, had been extensively researched [3–6]. While as another kind of important carotenoids, C_30_ carotenoids were seemingly ignored from the beginning.

Dendritic cells (DCs) are potent antigen-presenting cells that play a major role in the initiation and regulation of the immune response [7]. DCs are derived from bone marrow progenitor cells and exist at two different stages: immature and mature [8]. Immature DCs (iDCs) are efficient at capturing extracellular antigens. However, iDCs are poorly immunogenic as they express low levels of MHC II (major histocompatibility complex II) molecules and co-stimulatory receptors including CD80, CD86 and CD40 on the cells surface. In response to a spectrum of stimuli, iDCs undergo a maturation process by up-regulating the expression of MHCII and co-stimulatory molecules, as well as the production of cytokines, greatly enhancing their ability to active T cells [9].

Mitogen-activated protein kinases (MAPKs) are one of the most classic signal transduction pathways in physiological processes and have been reported to be involved in the DC maturation [10]. Many pro- or anti-inflammatory stimulus induce or inhibit phosphorylation of MAPKs, such as extracellular signal-regulated kinases (ERK), c-Jun N-terminal kinases (JNK), and p38 MAPK. The three MAPK signaling-pathways have distinct roles in the DC maturation process.

CD36 (cluster of differentiation 36) is a class B scavenger receptor that binds ligands of both pathogen and self-origin [11], which is capable of initiating intracellular signaling cascades that activate multiple genes, such as those encoding cytokines and co-stimulatory molecules [12–14]. On the other hand, CD36 involves in the absorption of C_40_ carotenoids in different cells [15, 16]. Based on the two points above, CD36 may be also related to carotenoids-derived intracellular signaling.

In this study, the functions of Dia, a C_30_ carotenoid, on DCs maturation were identified for the first time. The results indicated that Dia was a potent inducer of DCs maturation via CD36 and NF-κB signals in a reactive oxygen species (ROS) independent manner. Dia-treated DCs had a stronger ability to stimulate T cell proliferation and Th1 polarization. However, β-carotene had opposite effects on DCs phenotypic maturation and reduced IL-12p70 production. These findings provided new insight into the different immune functions of carotenoids. Moreover, due to the critical roles of these professional antigen presenting cells in the initiation and regulation of immune responses and the *Bacillus subtilis* (a probiotics) [17, 18]-derived feature of Dia, our findings might have important implications for the manipulation of DCs functions for potential therapeutic application.

## RESULTS

### Obtainment of 4,4′-diaponeurosporene (Dia) and toxicity analysis on DCs

**Figure 1:**
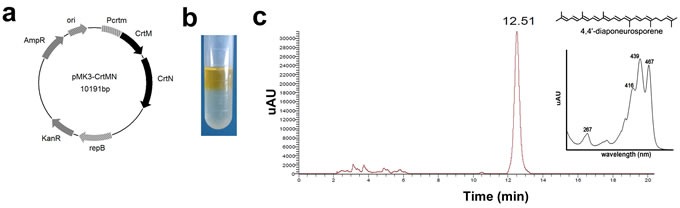
The biosynthesis, identification, and toxicity analysis of 4,4′-diaponeurosporene (Dia) **a**. Structure of the carotenoid-producing plasmid pMK3-*crtMN*. Pcrtm, promoter of crtm; AmpR, ampicillin-resistance gene; ori, replication origin; KanR, kanamycin-resistance gene; repB, replicon. **b**. The pigment in *Bacillus subtilis* (*B. subtilis*) harboring pMK3-crtMN could be extracted with hexane and dissolved in hexane layer. **c**. HPLC analysis of the pigment produced by *B. subtilis* harboring pMK3-crtMN. Retention time of the main peak and absorption maximum of the main peak are indicated. The chemical structure of Dia is also shown (from reference 20).

To obtain Dia, a plasmid (pMK3-crtMN) contained two carotenoid synthases, dehydrosqualene synthase, crtM and dehydrosqualene desaturase, crtN, was constructed (Figure 1a). The *Bacillus subtilis* (*B. subtilis*) harboring pMK3-crtMN appeared yellow, and the yellow pigment could be extracted with hexane (Figure 1b). The HPLC chromatogram of extract from *B. subtilis* harboring pMK3-crtMN showed one major peak, which was identical to those of 4,4′-diaponeurosporene (abbreviated to “Dia”) [19–21], at 12.51 min (Figure 1c). These results suggested that Dia was the main composition in the extract. As Figure 1c showed, there were several little peaks of other constituents before the peak of Dia. This indicated the extraction was not specific. To make the results more accurate, together with Dia, the immunologic functions of the extraction from *B. subtilis* harboring pMK3 was also evaluated, named as “Control Extract” (abbreviated to “CE”). The chemical structure of Dia was shown in Figure 1c [22].

Then, the relative cytotoxicity effect of Dia against DCs was assessed by the CCK-8 viability assay. The results exhibited that Dia revealed no cytotoxicity against DCs when the concentration was below 20 μM (data not displayed). As the concentration of 1 μM was estimated to be close to the physiological level of carotenoids [23], it was adopted as the experimental dose in the following experiments.

### Dia induced DCs maturation

**Figure 2:**
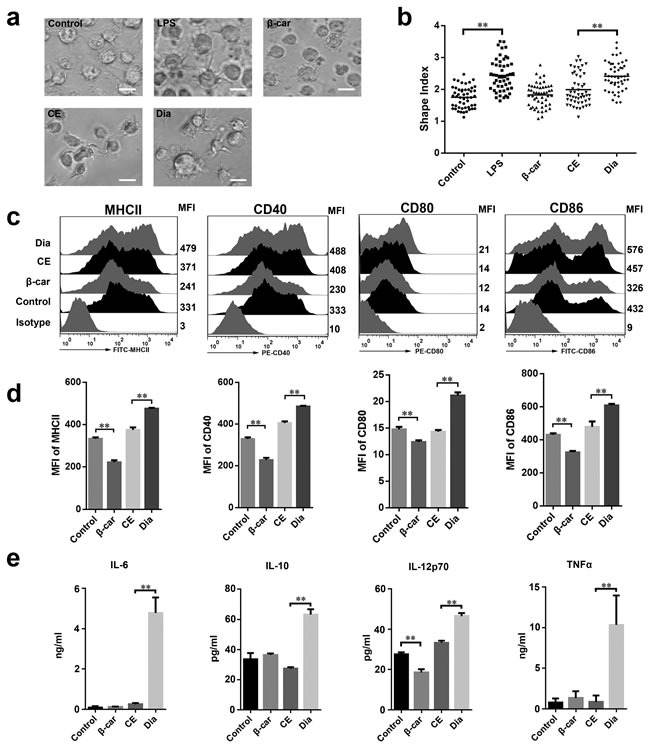
Dia induced dendritic cells (DCs) maturation **a**. DCs were treated with DMSO (equal volume to Dia, as control), LPS (10 ng/ml), β-carotene (β-car) (1 μM), CE (equal volume to Dia) or Dia (1 μM) respectively for 24 hours and morphology of DCs dendrites were observed by microscopy. Scale bar = 30 μm. **b**. The statistical result of the cellular shape index. Horizontal lines across the scatter diagram represent mean values. n = 50 (50 dendritic cells randomly selected from 3 separate experiments). **c**. DCs with different treatments were stained for the indicated surface molecules and analyzed by FACS. **d**. The mean fluorescence intensity (MFI) values of indicated surface molecules are shown as the mean ± SD. **e**. Supernatants of DCs culture were collected and tested for IL-6, IL-10, IL-12p70, and TNFα by ELISA. Data are represented as mean ± S.D. One representative of three similar independent experiments is shown.

Compared with immature DCs, mature DCs easily form longer extensions [24–26]. So, to investigate the role of β-carotene and Dia in modulating DCs functions, the morphological changes of DCs stimulated by β-carotene and Dia were detected firstly. Same with lipopolysaccharide (LPS), a mature positive control, Dia could lengthen dendrites and increase the shape index of DCs, while the same concentration of β-carotene had no significant influence on DCs morphological maturation (Figure 2a, 2b). Moreover, to confirm this result, we assessed the phenotype maturation of DCs by FACS. The result showed that, as well as CD40, CD80 and CD86, the expression of MHCII was significantly enhanced by Dia, while β-carotene reduced the expression of these phenotypes (Figure 2c, 2d). These indicated that Dia induced the morphological and phenotypic maturation of DCs, but β-carotene had an opposite role.

Mature DCs secrete a variety of pro- and anti-inflammatory cytokines and play vital roles in regulation of immune responses. To determine whether Dia and β-carotene could have influences on cytokines produced by DCs, the secretion of IL-6, IL-10, IL-12p70 and TNFα by DCs with different treatments were assessed. As expected, Dia increased the production of all these four cytokines, while β-carotene reduced the secretion of IL-12p70 and had no significant influence on other three cytokines (Figure 2e).

### Dia efficiently enhanced DCs antigen capture in vitro and increased the number of antigen-loaded DCs in mesenteric lymph nodes (MLN)

**Figure 3:**
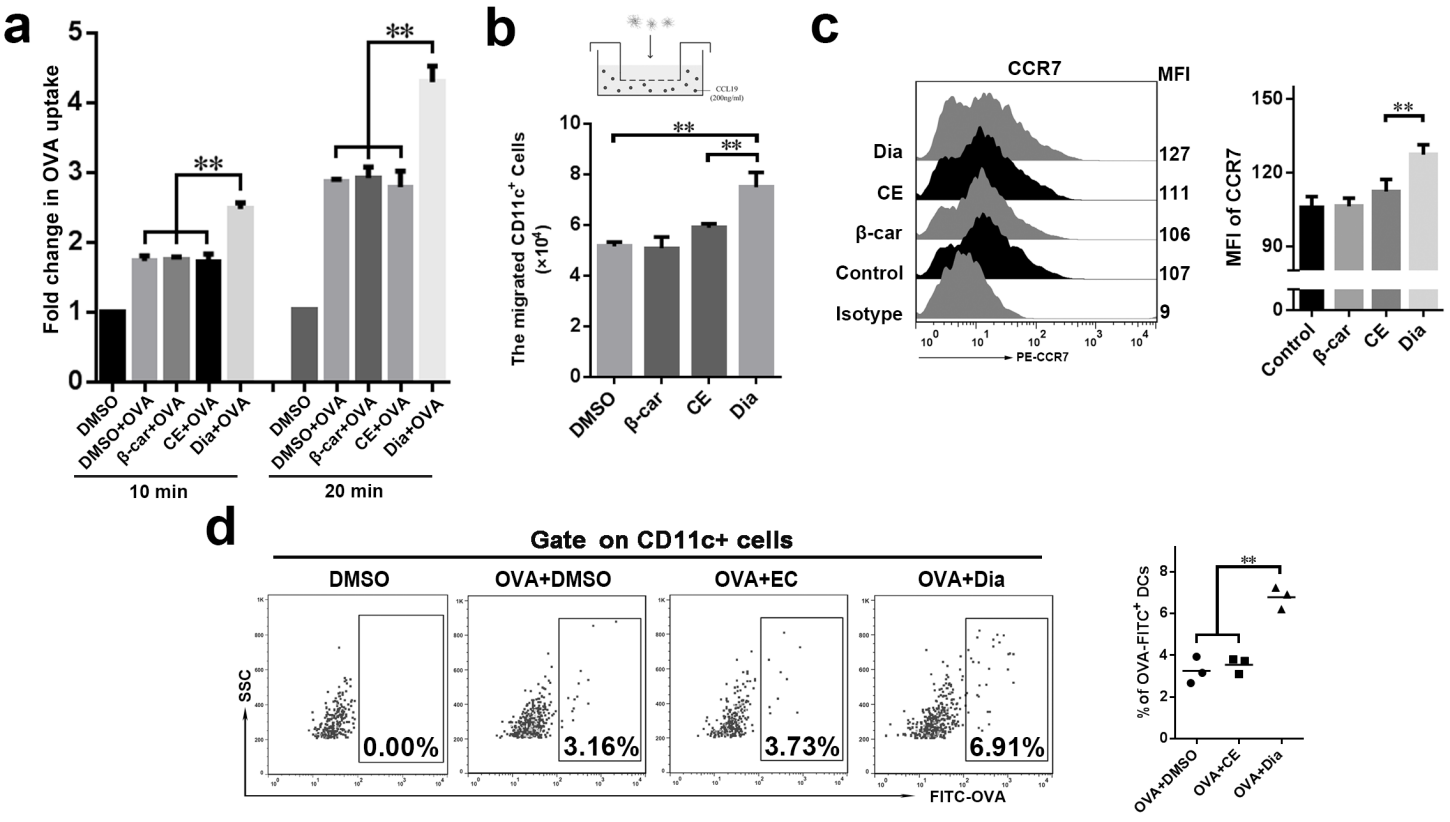
Dia efficiently enhanced DCs antigen capture *in vitro* and increased the number of antigen-loaded DCs in mesenteric lymph nodes (MLN) **a**. The antigen uptake of DCs incubated with 1 μg/ml FITC-OVA combined with DMSO (equal volume to Dia), β-carotene (1 μM), CE (equal volume to Dia) or Dia (1 μM) respectively were analyzed by flow cytometry. The average uptake of DCs incubated with FITC-OVA at 4°C was taken as 1 (shown as the “control” group). **b**. DCs were seeded into the upper wells of a 24-well transwell chamber, and CCL19 (200 ng/ml) was included in the lower wells. After 4 hours, the number of DCs that transferred from the upper to the lower wells was counted. **c**. The expressions of CCR7 were determined by FACS. **d**. Mice terminal ileal ligated loop was injected with DMSO or 100 μl FITC labeled OVA (10 μg/ml) containing Dia, DMSO or CE. 2 hours later, the OVA-loaded DCs in MLN were analyzed by FACS. Data are represented as mean ± S.D. One representative of three similar independent experiments is shown.

As one of the most important antigen presenting cells, DCs have the ability to capture antigens and migrate to lymphoid organs to active T cells [27, 28]. This process is necessary for inducing immune responses. So, the influences of Dia on antigen capture and migration ability of DCs *in vitro* and/or *in vivo* were evaluated. The result showed that DCs treated with Dia had an enhanced antigen capture ability, on which β-carotene had no significant influence (Figure 3a).

We next asked if Dia could influence the chemotactic function of DCs. DCs migration was evaluated using chemotaxis assay in transwell chambers on the basis of attraction of mature DCs for chemokine (C-C motif) ligand 19 (CCL19). The migration of Dia-treated DCs was remarkably enhanced in response to CCL19, while that of β-carotene-stimulated DCs was not (Figure 3b). Then the expression of C-C chemokine receptor type 7 (CCR7), the receptor of CCL19, was examined. As expected, Dia increased the expression of CCR7 on DCs (Figure 3c). Thus, these data indicated that Dia promoted DCs migration.

For further study, mouse intestinal ligature test was performed. The fluorescein isothiocyanate (FITC) labeled ovalbumin (OVA) combined with DMSO, EC or Dia respectively, was injected into the ligated loops of C57BL/6 mice. We found that Dia could remarkably increase the number of ovalbumin loaded DCs in MLN (Figure 3d), which suggested it could enhance migration and/or antigen capture ability of DCs.

### Dia-treated DCs promoted T cell proliferation and Th1 polarization

**Figure 4:**
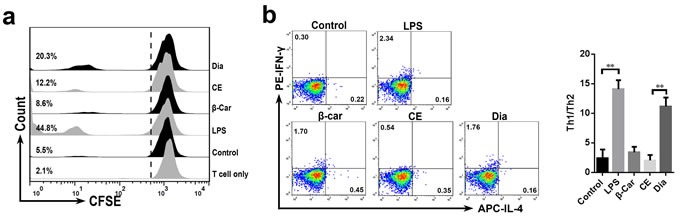
Dia-treated DCs promoted T cell proliferation and Th1 polarization **a**. DCs were incubated with β-carotene (1 μM), CE (equal volume to Dia), Dia (1 μM), or LPS (10 ng/ml) respectively for 24 hours. cells were washed extensively and then co-cultured with CFSE-labeled T cells at a ratio of 1:10. Control represents unstimulated DCs. Single T cell group is also shown. T cell proliferation was analyzed by CFSE dilution using FACS. Percentages refer to proportion of T cells that proliferated within 5 days. **b**. DC-driven Th1/Th2 differentiation was detected. DCs with different treatments were incubated with autologous naïve CD4^+^ T cells at a ratio of 1:4 for 15 days. LPS-stimulated DCs co-cultured with naïve T cells were as a positive control. The resulting T cells were analyzed for their capacity to secrete IFN-γ and IL-4. Data are represented as mean ± S.D. One representative of three similar independent experiments is shown.

After arriving at mesenteric lymph nodes, DCs will interact with T cells and induce immune responses. So next we investigated the potential of Dia to prime T cells responses after its interactions with DCs. As the result showed, Dia-treated DCs had an increased ability to promote T cell proliferation (Figure 4a), while that of β-carotene-stimulated DCs had no significant changes.

Since Dia could enhance the allostimulatory capacity of DCs, we wondered if Dia also influenced the T cell polarization by affecting DCs. So, an experiment of DC-driven Th1/Th2 differentiation was performed. In agreement with previous studies [29–31], DCs incubated with LPS induced naïve T cell differentiation into a Th1 response. We also found that Dia induced more IFN-γ, marker for Th1, compared with IL-4 producing T cells (Figure 4b). These results demonstrated that Dia-treated DCs promoted Th1 polarization.

### Dia induced the phosphorylation of mitogen-induced protein kinases (MAPKs) and the activation of NF-κB in a ROS-independent manner

**Figure 5:**
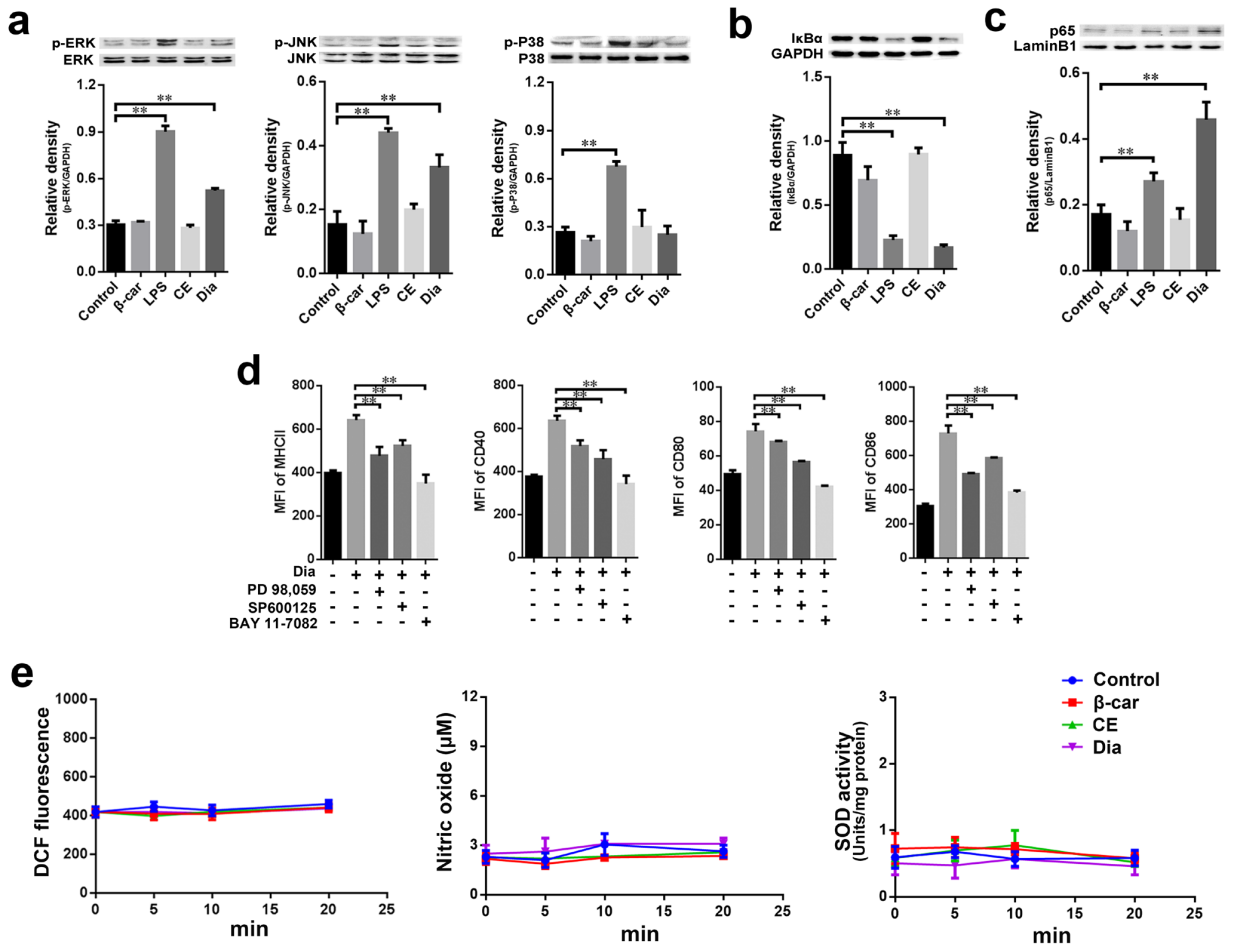
Dia induced the phosphorylation of MAPKs and the activation of NF-κB without changing the redox state of DCs **a.-c**. DCs were treated with β-carotene (1 μM), LPS (10 ng/ml), CE (equal volume to Dia) or Dia (1 μM) respectively for 30 min, and the phosphorylation of ERK, JNK, P38 (a) IκB-α (b) and nuclear p65 (c) were detected by western blot. **d**. The inhibitors of ERK, JNK, and NF-κB signals markedly impaired Dia-induced DCs maturation. DCs were pretreated with different inhibitors in the concentration of 10 μM for 2 hours, then treated with Dia for another 24 hours. DCs were stained for the indicated surface molecules and analyzed by FACS. **e**. Dia and β-carotene had no significant influence on the redox state of DCs in normal condition. β-carotene (1 μM), CE (equal volume to Dia) or Dia (1 μM) were added into DCs culture respectively. At different time points, cells were harvested. For ROS, cells were stained with DCFDA and analyzed by FACS. For nitric oxide and superoxide dismutase, cells were detected by nitric oxide or superoxide dismutase assay kits. Data are represented as mean ± S.D. One representative of three similar independent experiments is shown.

MAPKs and NF-κB activation are important events underlying DCs maturation [10, 32–34]. To gain insight into Dia-induced DC maturation, we determined whether MAPKs and NF-κB signals might be involved in Dia-induced signaling pathways in DCs. Lots of studies indicated that LPS could activate MAPKs and NF-κB in DCs [35–37], so, here we employed LPS as a positive control. The results showed that Dia enhanced the phosphorylation levels of ERK and JNK, but not p38, while β-carotene had no notable influences on the phosphorylation of MAPKs (Figure 5a).

Next, to detect the activation level of NF-κB, the degradation of IκB-α and the nuclear localization of p65 were evaluated. The results showed that Dia promoted the two indexes mentioned above, but β-carotene had no remarkable effect (Figure 5b, 5c, Supplementary Figure 1a). Then PD98, 059 (inhibitor of ERK activation), SP600125 (inhibitor of JNK activation) and BAY 11-7082 (inhibitor of NF-κB), were employed to confirm ERK, JNK and NF-κB signals in Dia-induced DCs maturation. The results showed that all these three inhibitors could inhibit Dia-induced phenotypic maturation of DCs (Figure 5d), further confirming that ERK, JNK and NF-κB signals played important roles in Dia-induced DCs maturation.

Considering the potential reducibility of Dia and the importance of reactive oxygen species (ROS) in signal transduction [38], we detected the reducibility of Dia and the level of ROS in DCs treated with Dia. Both Dia and β-carotene could reduce the hydrogen peroxide induced oxidative stress in DCs (Supplementary Figure 1b, 1c), but had no significant influence on ROS in normal condition (Figure 5e). To more comprehensively evaluate DCs redox state, we also detected the levels of nitric oxide (NO) and superoxide dismutase (SOD) in Dia-treated DCs. Consistent with ROS, no significant changes were observed (Figure 5e). So these results suggested that redox signals might not be employed by Dia to activate ERK, JNK and NF-κB.

### CD36 had a critical role in Dia-induced DCs maturation

**Figure 6:**
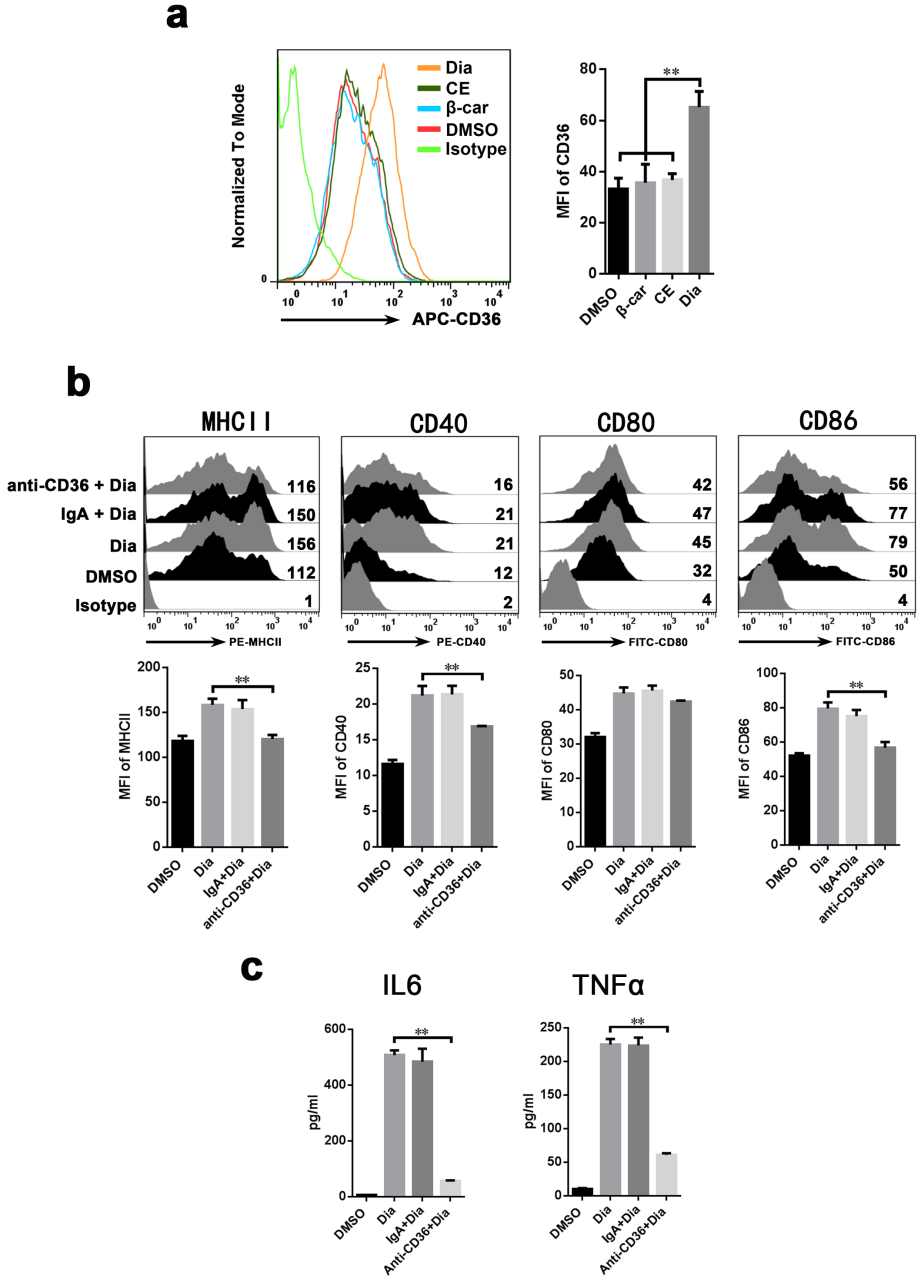
Antibody against CD36 inhibit the Dia-induced maturation of DCs **a**. Dia treated DCs had an up-regulated expression of CD36. DCs were treated with DMSO (equal volume to Dia), EC (equal volume to Dia) or Dia (1 μM) for 24 hours, then stained for CD36 and analyzed by FACS. **b**. DCs were exposed to control IgA, anti-CD36 mAbs (IgA) at the concentration of 30 μg/ml for 1 hour, followed by adding Dia (1 μM). 24 hours later, DCs were stained for the indicated surface molecules and analyzed by FACS. **c**. DCs was treated as described in (b). The supernatants were collected and tested for IL-6 and TNFα by ELISA. Data are represented as mean ± S.D. One representative of three similar independent experiments is shown.

CD36 is involved in the absorption of carotenoids and the function of DCs [39]. Moreover, we found that Dia-treated DCs had an up-regulated expression of CD36, while that of CE and β-carotene had no significant change (Figure 6a). So, it is likely that CD36 participates in Dia-induced DCs maturation. To validate this hypothesis, CD36 on DCs was blocked by antibody before Dia treatment, and the phenotypes of DCs was detected by FACS. We found that blocking of CD36 impaired Dia-induced DCs maturation by significantly down-regulate the expression of MHCII, CD40 and CD86 (Figure 6b), and this was also accompanied by a reduced ability to release cytokines (Figure 6c). The expression of CD80 also had the tendency to reduce, though non-significant (*P* = 0.06) (Figure 6b). In conclusion, these results indicated the critical role of CD36 in Dia-induced DCs maturation.

## DISCUSSION

This was the first report of which we were aware concerning the effects of C_30_ carotenoid on the functions of DCs. In previous studies, many researches have reported that C_40_ carotenoids exerted an anti-inflammatory property [40–42], and could reduce LPS-induced activation of macrophages through the inhibition of the NF-κB signal pathway [43–45]. Nevertheless, the effect of β-carotene on the maturation and immunological responses of DCs largely remains unknown. More seriously, we almost have no knowledge about the immune functions of C_30_ carotenoids.

DCs are considered to play a key role in the establishment of both innate and adaptive immune responses [46], and the maturation is responsible for the ability of DCs to tailor immune responses [8]. Therefore, we performed functional assays to ascertain the phenotypes and functions of DCs treated with Dia and β-carotene. We found the effect of Dia primarily involved the activation of CD36 signal as well as MAPKs and NF-κB p65. These results indicated that Dia was conclusively a potent inducer of DCs maturation, while β-carotene seemed to have an inhibiting effect. It is a generally accepted notion that activation of the immune system may be a double edged sword. The activation of immune system could provide protection against pathogens, while the accompanied inflammatory aspects may also aggravate certain chronic diseases. Consider the different roles of Dia and β-carotene on DCs maturation, they might have respective applications in different immune states.

Accumulating evidences suggests that cytokine production of DCs depends on stimuli that DCs receive [47]. Especially, IL-12 has multiple immuneregulatory functions, including the activation of Th1 subset, which plays a pivotal role in the resistance of intracellular parasitic microorganisms [48]. It has also been reported that β-carotene inhibited IL-12p40 expression of macrophages [45], and lycopene, another C_40_ carotenoid, inhibited IL-12 secretion of DCs [44]. However, the effects of Dia and β-carotene have not previously been shown on DCs. In the present study, we showed that β-carotene also had an inhibitory function on DCs phenotypic maturation and IL-12p70 production. However, Dia induced DCs to produce IL-12, as well as other pro-inflammatory cytokines (IL-6, and TNFα), indicating that Dia had different influences on DCs from C_40_ carotenoids β-carotene and lycopene. Importantly, Dia also induced DCs to produce IL-10, a anti-inflammatory cytokine. This was very important to prevent inducing an excessive inflammatory response.

A line of evidences indicate that the development towards Th1 cells is regulated mainly by DC-derived cytokines, such as IL-12 [49]. We found Dia could enhance the capacity of DCs to initiate Th1 responses *in vitro*. Since Th1 cells are functionally immunogenic and protective against invading pathogens, the promotion of DC-mediated Th1 polarization may be benefit for establishing immune responses against virus. Moreover, the induction of Th1 development exerts a positive regulatory role for a wide variety of immune cells [50]. Hence, the induction by Dia of Th1 polarization might contribute to the induction of the immunoenhancement state.

It is well recorded that ROS are created by a variety of cellular processes as part of cellular signaling events and have various inhibitory or stimulatory roles in NF-κB signaling [38, 51, 52]. As a well-known antioxidant, C_40_ carotenoids were considered to prevent oxysterol-induced inflammation in macrophage by compromising the MAPKs and NF-κB signals [53]. In our study, both β-carotene and Dia represented antioxidant properties under the situation of hydrogen peroxide-induced oxidative stress, but both of them could not change the redox signals in normal condition. These results indicated that ROS might not be the agent of Dia-induced activation of ERK, JNK MAPKs and NF-κB signals.

It has been well accepted that MAPKs and NF-κB signaling pathways play important roles in the maturation and pro-inflammatory cytokines expression of DCs. The data from experimental studies have demonstrated that β-carotene [43], lutein [4], and lycopene [54] inhibit activation of MAPKs and/or NF-κB, suggesting the inhibitory effects of C_40_ carotenoids on DCs maturation. While, different from β-carotene, Dia could effectually active ERK, JNK MAPKs and NF-κB signals, leading to the maturation of DCs. This brought light to the mechanism underlying the contrary effects of C_30_ carotenoids and C_40_ carotenoids on DCs maturation. Moreover, Dia induced a high expression of CD36, which could inhibit DCs maturation when blocked. Considering the important roles of CD36 in activation of MAPKs, CD36 might be served as a linker between Dia and MAPKs.

In conclusion, we revealed β-carotene inhibited the phenotypic maturation and reduced the IL-12p70 production of DCs, while Dia induced DCs maturation and increased the cytokines production via CD36, MAPKs and NF-κB signaling, resulting in the promotion of Th1 development. In addition, Dia could enhance antigen capture ability of DCs and increase frequency of antigen loaded DCs in MLN, and might be in favor of inducing immune responses to antigens in intestinal tract. These findings underpinned the different roles of C_30_ and C_40_ carotenoids on DCs maturation for the first time. Though the reason leading to this phenomenon is not clear, their differences in molecular structures or metabolic processes in DCs might be a potential cause. Moreover, the Dia derived from *Bacillus subtilis*, a probiotic, it might have a possibility of application in immune-compromised individuals.

**Figure 7:**
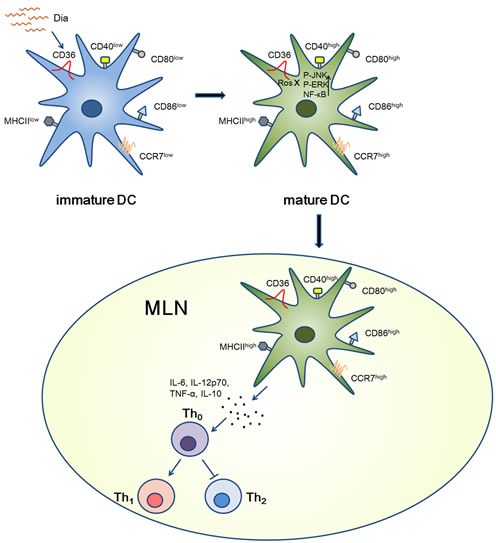
Graphical Abstract Schematic view of Dia-induced DCs maturation. When encountered with immature DCs, Dia could induce DCs maturation by up-regulating the expression of MHCII and other co-stimulatory molecules via CD36 and NF-κB signals. As the same time, the expression of CCR7 was also up-regulated, resulting in a high migration ability towards MLN. After arriving at MLN, DCs promoted Th1 polarization.

## MATERIALS AND METHODS

### Animals

C57BL/6 mice, 4-6 weeks old, were from the Animal Research Center of Yangzhou University (Jiangsu, China). The mice were maintained under specific pathogen free conditions for at least 1 week before use. The animal studies were approved by the Institutional Animal Care and Use Committee of Nanjing Agricultural University and followed National Institutes of Health guidelines for the performance of animal experiments.

### Reagents

Fluorescent-labeled anti-mouse mAbs APC-CD11c (N418), PE-CD40 (1C10), PE-CD80 (16-10A1), FITC-CD86 (GL1), FITC-MHCII (M5/114.15.2), PE-CCR7 (4B12), FITC-CD4 (GK1.5), APC-IL-4 (11B11), PE-IFN-γ (XMG1.2), or respective isotype controls were purchased from eBioscience (San Diego, CA, USA). Mouse CD36 blocking antibody [JC63.1] and control IgA antibody were from Abcam (Cambridge, USA). Rabbit anti-mouse pERK, ERK, pJNK, JNK, pP38, P38, IκBα, p65, LaminB1, GAPDH, goat anti-rabbit IgG-DY488 and goat anti-rabbit IgG-HRP were from Bioworld (St. Louis Park, MN, USA). RPMI 1640 medium, DAPI, FITC-OVA, and CFSE were from Invitrogen (Grand Island, NY, USA). Fetal bovine serum (FBS) was from Hyclone (Thermo, Melbourne, Australia). Recombinant GM-CSF, IL-4, IL-2, and CCL19 were from Peprotech (Rocky Hill, NJ). LPS (from Escherichia coli 026: B6), 2′,7′-dichlorodi hydro fluorescein diacetate (DCFDA) and β-carotene were from Sigma-Aldrich (St Louis, MO, USA). PD 98, 059, SP600125, BAY 11-7082, Bradford assay kit, Nitric Oxide assay kit, Superoxide dismutase assay kit, and Nuclear and Cytoplasmic Protein Extraction Kit were from Beyotime (Jiangsu, China). Mouse IL-6, IL-10, IL-12p70, TNFα ELISA Kit and Cell Counting Kit-8 (CCK8) were from Boster (Wuhan, China).

### Bacterial strains and growth conditions

*B. subtilis* 168 purchased form Hangzhou Biosci Biotech Company (Hangzhou. China). *Staphylococcus aureus* ATCC25923 and *E. coli* DH5α were used for genetic construction. All bacteria strains were grown in Luria-Bertani (LB) broth (10 g tryptone, 5 g yeast extract, and 5 g NaCl per liter) or on LB plates fortified with 1.5% agar at 37°C. Appropriate antibiotics were included at the following concentrations: 50 μg/ml kanamycin and 100 μg/ml ampicillin.

### Construction of carotenoid-producing plasmid and electroporation

The *B. subtilis-E. coli* shuttle vector, pMK3, was used to construct the carotenoid-producing plasmid. The total DNA was extracted from *S. aureus* cells using the DNeasy tissue kit (Qiagen, Duesseldorf, Germany). The fragment containing the coding sequence of crtM, crtN and the promoter was amplified from the total DNA of *Staphylococcus aureus* using the primers obtained from Invitrogen (Life Technology, Shanghai, China), 5′-TGATTAC GCCAAGCTGCATTTGGACCGGTGACATTAACAA 3′ and 5′-GAATTCCCGGGGATC TTAAGCGTAATCTGGAACATCGTATGGGTATACG - 3′ (the homologous sequences were underlined), and cloned into the pMK3 vector digested with *Hind III* and *BamHI*, following the instructions of ClonExpress^TM^ II One Step Cloning Kit (Vazyme Biotech, Nanjing, China), generating the carotenoid-producing plasmid pMK3-crtMN.

Electroporation of *B. subtilis* was carried out according to the HO method described by Yang. *et al*. [55], with minor modifications. Briefly, *B. subtilis* was grown in growth medium (LB containing 0.5 M sorbitol) at 37°C to OD600 reached 1.0. Then, cells were harvested by centrifugation at 4°C and 5,000 g for 8 min, following four washes in ice-cold EP buffer (0.5 M sorbitol, 0.5 M mannitol and 10% glycerol). Finally, the cells were suspended in 1/40 of the culture volume of the electroporation medium with EP buffer. 60 μl of competence cells were mixed with 2 μg DNA, then transferred the mixture to an electroporation cuvette (0.1 cm electrode gap) and the cells were exposed to a single electrical pulse (2,200 V, 25 μF) using Multiporator 4308 (Eppendorf, Germany). Immediately following the electrical discharge, 1 ml of recovery medium (LB containing 0.5 M sorbitol and 0.38 M mannitol) were added into the cells. After incubated at 37°C for 3 hours, cells were plated on LB-agar plates with kanamycin antibiotic for screen.

### Extraction of carotenoids

The extraction of carotenoids was carried out according to the methods described previously [21, 56], with minor modifications. A single colony of transformant was inoculated into LB medium and cultured for 24 hours. Cells in 1 L LB medium were collected by centrifugation and washed with 20 ml Tris-EDTA (TE) buffer (10 mM Tris/HCl, 1 mM EDTA, pH 8.0), then re-suspended in 4 ml TE buffer. 1 ml of 20 mg/ml lysozyme was added and incubated for 30 min at 37°C. The cell lysate was then transferred into a glass tube, and 10 ml acetone was added. The mixture of acetone and cell lysate was heated for 2 min in a water bath at 55°C, and then vortex-mixed for 1 min. The extract was transferred into a new glass tube. The acetone extraction was repeated three times. The result extracts were re-extracted with hexane after addition of 1/2 volume of saltwater, and dried under nitrogen gas, then the pigment sediment was weighed and redissolved into 1 ml DMSO and stored at −70°C for use.

### HPLC

The pigment extract was filtered and analyzed with a Thermo HPLC system (Thermo, SURVEYOR MS Pump Plus) equipped with a μBondapak C18 column (10 μm particle size, 3.9 × 300 mm, 125 A° pore size, Waters). 1 μl pigment solution was eluted with acetonitrile/water (90 : 10, v/v) at 200 μl/min. The spectrum from 200 nm to 800 nm was monitored with SURVEYOR PDA Plus Detector. Pigment was identified from their absorption spectra.

### Generation of DCs

DCs were generated as previously reported [57]. Briefly, bone marrow cells of C57BL/6 mice (4 weeks old) were flushed from the tibias and femurs and cultured in complete medium (RPMI1640 with 10% FBS, 1% streptomycin and penicillin, 10 ng/ml GM-CSF and IL-4). On day 3, medium was gently replaced with fresh medium. On day 6, non-adherent and loosely adherent DCs aggregates were harvested and subcultured overnight. On day 7, 90% or more of the CD11c^+^ non-adherent cells were used.

### Phenotype assay by FACS

DCs were stimulated with Dia (1 μM), CE (equal volume to Dia), β-carotene (1 μM) or LPS (10 ng/ml) for 24 hours, then washed twice with cold PBS and stained with the fluorescent mAbs specific for mouse CD11c, CD40, CD80, CD86, and MHCII, or the respective isotype controls at 4°C for 30 min as per the manufacturer's guidelines. After washing three times with PBS, the cells were phenotypically analyzed by FACS (BD FACSCalibur).

### Cytokine assays by enzyme-linked immunosorbent assay

DCs were stimulated with Dia (1 μM), CE (equal volume to Dia), β-carotene (1 μM) or LPS (10 ng/ml) for 24 hours. The cytokines (IL-12p70, IL-10, IL-6 and TNFα) in culture mediums were measured using enzyme-linked immunosorbent assay (Boster, Wuhan, China), and performed according to the manufacturer's instructions.

### Migration assay

Migration assay was performed as described previously [58]. BMDCs in serum-free medium were placed in a 24-well transwell migration chamber (pore size, 8 μm; Corning, NY, USA). 0.1 ml RPMI 1640 medium containing DCs (1 × 10^5^ cells) was loaded onto the upper wells. 0.6 ml RPMI 1640 medium containing CCL19 (200 ng/ml) was added to the lower chambers to induce cell chemotaxis. After incubation for 4 hours at 37°C, the migrated cells were collected from the lower wells, and the number of CD11c^+^ cells was determined by FACS.

### Autologous mixed lymphocyte reaction and the DC-driven Th1/Th2 differentiation

The functional activity of DCs was reflected in the primary autologous mixed lymphocyte reaction assay. DCs were stimulated with Dia (1 μM), CE (equal volume to Dia), β-carotene (1 μM) or LPS (10 ng/ml) for 24 hours, washed and incubated with CFSE labeled responder T cells, purified from mesenteric lymph node using T cell isolation kit (Miltenyi Biotech, Germany), at a rate of DC : T = 1 : 10. Five days later, the proliferation of T cell was detected by FACS. For the Th1 or Th2 differentiation, DCs were stimulated with Dia, CE, β-carotene or LPS as described above, and incubated with autologous naïve CD4^+^ T cells as described by Sergey R [59]. DCs stimulated with LPS were co-cultured with naïve T cells as a positive control. At day 5, 10 U/ml IL-2 was added. All co-cultures of DCs and naïve T cells were performed for up to 15 days, and the resulting T cells were analyzed for their capacity to secrete IFN-γ and IL-4, markers for Th1 or Th2 differentiation, respectively.

### *In vitro* antigen uptake assay

DCs were left untreated or incubated with 1 μg/ml FITC labeled ovalbumin (FITC-OVA) combined with DMSO (equal volume to Dia), β-carotene (1 μM), CE (equal volume to Dia) or Dia (1 μM) at 4°C or 37°C for 10 min or 20 min. After washing with PBS, the FITC-OVA uptake was analyzed by FACS.

### Ligated loop experiments

C57BL/6 mice (8 weeks old) were pretreated with broad-spectrum antibiotics for 5 days and anesthetized with chloral hydrate (350 mg/kg body weight, intraperitoneal). Mice Terminal ileal ligated loop was injected with DMSO (equal volume to Dia), FITC labeled OVA (10 μg/ml), OVA plus Dia (1 μM) or OVA plus CE (equal volume to Dia), respectively. After 2 hours, MLNs were collected and the OVA loaded DCs in MLN were analyzed by FACS.

### Western blotting

Cells were rinsed twice with cold PBS, and solubilized in lysis buffer containing 50mM Tris-HCl, pH 7.6, 150 mM NaCl, 1 mM ethylenediaminetetraacetic acid (EDTA), 1% (m/v) NP-40, 0.2 mM phenylmethylsulfonyl fluoride (PMSF), 0.1 mM NaF and 1.0 mM dithiothreitol (DTT) for 30 min on ice. Lysates were centrifuged (16000 g) at 4°C for 10 min, and the protein concentrations in supernatants were measured using a Bradford assay kit (Beyotime, China). To detected p65 nuclear translocation, the nuclear protein was extracted follow the instructions of Nuclear and Cytoplasmic Protein Extraction Kit. Equal amounts of the soluble protein were denatured in sodium dodecyl sulfate (SDS), electrophoresed on a 12% SDS-polyacrylamide gel, and transferred to polyvinylidene fluoride (PVDF) membranes. Then blocked for non-specific binding in blocking buffer (5% bovine serum albumin (BSA) in TBST (Tris Buffered Saline With Tween, sodium chloride 8.8 g, 1 M Tris-HCl 10 ml, Tween-20 1 ml, ddH_2_O fill to 1 L)) for 2 hours. Subsequently, the membranes were incubated with antibodies overnight and exposed to HRP-conjugated secondary antibody for 2 hours. Protein bands were visualized with enhanced chemiluminescence (ECL) Western blotting detection reagents. The ECL image was recorded using the FluorChem Xplor (Alpha Innotech, U.S.A.), and the optical density of an equal surface area for each band was determined using Image J software. All blots were stripped and re-probed with polyclonal anti-GAPDH or LaminB1 antibodies to a certain equal loading of proteins.

### Determination of ROS production

ROS production in DCs was determined utilizing 2′,7′-dichlorodihydro fluorescein diacetate (DCFDA). Briefly, 5 × 10^5^ cells/ml were taken in a culture dish and treated with DMSO (equal volume to Dia, as control), Dia (1 μM), CE (equal volume to Dia) or β-carotene (1 μM) for 0, 5, 10, or 20 min. Then, cells were collected and DCFDA was added into cell suspensions at a final concentration of 10 μM. After 30 min of incubation in the dark at 37°C, cells were centrifuged and the pellets were washed twice with ice-cold PBS. Cells were re-suspended in FACS buffer and the fluorescence was analyzed with FACS.

### Determination of nitric oxide (NO) and superoxide dismutase (SOD)

Briefly, 5 × 10^5^ cells/ml were taken in a culture dish and treated with DMSO (equal volume to Dia, as control), Dia (1 μM), CE (equal volume to Dia) or β-carotene (1 μM) for 0, 5, 10, or 20 min. Then, the NO and SOD in dendritic cells were measured using NO or SOD assay kits according to the directions.

### Statistical analysis

Results were expressed as means ± S.D. One-way ANOVA was employed to determine statistical differences among multiple groups and t test was employed to determine that between two groups. *P* values < 0.05 were considered significant (*P < 0.05, **P < 0.01).

## SUPPLEMENTARY FIGURE


