# Supplementary material for: 4,4′-diaponeurosporene, a C30 carotenoid, effectively activates dendritic cells via CD36 and NF-κB signaling in a ROS independent manner
Source: Oncotarget. 2016 Jun 2;7(27):40978–91. doi: 10.18632/oncotarget.9800 (PMC5173036; doi:10.18632/oncotarget.9800)
Supplement: Supplementary file 1 [file oncotarget-07-40978-s001.pdf]

4,4′-diaponeurosporene, a C_{30} carotenoid, effectively activates dendritic cells *via* CD36 and NF-κB signaling in a ROS independent manner

Supplementary Material

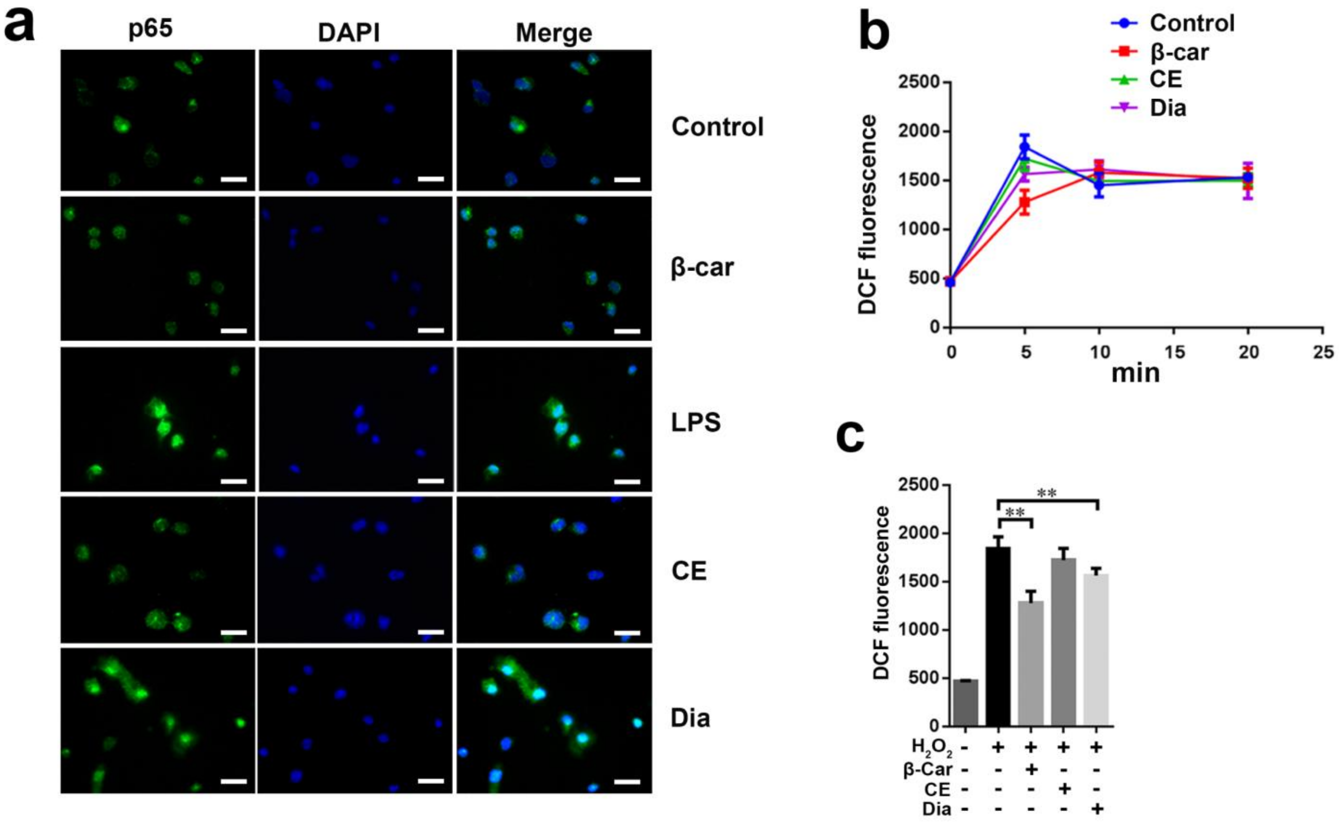

Supplementary Fig. 1 Dia induced the phosphorylation of MAPKs and the activation of NF-κB in a ROS-independent manner.

(a)The activation of NF-κB by Dia was also testified by the nuclear translocation of p65. p65 (green), DNA (blue). Scale bar = 30 μm. (b) Dia and β-carotene could reduce the hydrogen peroxide induced oxidative stress in DCs. Hydrogen peroxide alone or combined with β-carotene (1 μM), CE (equal volume to Dia), or Dia (1 μM) were added into DCs culture. At different time points, cells were harvested and stained with DCFDA and analyzed by FACS. (c) The statistical result at 5 min point in (f) is shown.
